# Spiritualism

**Published:** 1854-04

**Authors:** 


### REVIEW DEPARTMENT.

### ARTICLE XI.

SPIRITUALISM. By JOHN W. EDMONDS, and GEO. T. DEXTER, M. D., *with an Appendix, by* NATHANIEL P. TALLMADGE, *late U. S. Senator, and Governor of Wisconsin; "now concerning Spiritual Gifts, &c.," vol. i.* New York, PARTRIDGE & BRITTAN, 1853.

THOSE perfectionists who have been in the habit of congratulating themselves, their friends, and the human race generally, on the steady advance of the species to a high and pure intellectuality, must be a little staggered by recent developments. We have been so long entertained by self-complacent sneers at the dark ages, when people shuddered at white ladies that hovered over lonely wells, and grew mute with terror as the wild clangor of the spectral huntsman disturbed the quiet of the midnight sky, that we have almost arrived at the conclusion that we are superstition-proof. What have we in this glorious, this enlightened, this highly esteemed and highly be*steamed*, this over-lauded, over-worked, over-acted, and over-done nineteenth century—what have we to do with Salem witcheries and pious butcheries, and hobgoblins, the terror of our ancestors? Oh no! it was our fathers' task to stone the prophets, to us has been reserved the more congenial occupation of garnishing the sepulchres of the righteous. We are the philosophical, we are the reflecting, we are the enlightened, we are the progressive end of humanity. We make steamboats, and build bridges, and found empires, and tame lightning, and imprison light.—We are in the scientific line which is fast excluding the theological. We are great investigators, profound philosophers, and our physical science is to revolutionize not only the world, but the heart, soul and mind of man.

This science of ours is to squeeze superstition out of mankind with as much ease as a steam hydraulic press might be supposed to squeeze lemons for punch. All that is necessary is, to wave our wand, and "hey, presto, change!" this dull and savage multitude of men becomes refined, intelligent, moral and religious. The difficulty is, that every perfectionist has his own particular conjuring stick, but it is generally agreed on all hands among this large class of that portion of the race which has had the inestimable privilege to date its birth in 18—, that the species is advancing, though each, of course, attributes it to his own particular perfection-machine.

Such is the perfectionist twaddle to which we have all been compelled to listen for many long years past. We have hardly had the audacity to suspect that there could be any superstition left in the world. For what could be the function of Mr. Snooks, the peripatetic phrenologist and friend of women's rights, or Mr. Smith, the ontologist and lecturer on all subjects before young ladies' schools and young gentlemen's lyceums, if not to drive all superstition from the face of the earth, and especially from the presence of our self-styled refined and intellectual society?

Unfortunately, however, at the very crisis of perfection, there breaks out, directly under the nose of Messrs. Snooks and Smith, a superstition more contemptible, more disgusting, than any that has preceded it. This nineteenth century of ours, all perfection as it is, has begotten a monster as pernicious as any that the wild fancies of Mediæval barbarism has given to the world. It differs in one respect from the superstition of older days; it is destitute of the poetry which gilded the sombre edges of that intellectual cloud. In many of those old stories, there was often a touching pathos, a delicate though fantastic beauty, a grotesque grandeur, an unobtrusive but pure morality, which almost reconcile us to the mental degradation that would believe them. In this, there is only a bald, vulgar stupidity. You recognize its origin. It could have been engendered of no other brains than those of a set of sharp, sly swindlers, to whose imagination the narrow circle of a gold dollar was horizon enough. They have unmistakably stamped the monster superstition of the nineteenth century with their own image and superscription, and sent it forth into the world a naked cheat, devoid of poetry, romance or beauty of any kind—disgraceful in its origin, contemptible in its machinery, disgusting in its development.

The thing originated some few years ago with two sharp speculating women in New York, called, most appropriately, Fox and Fish. These females, of whose antecedents we know nothing, but of whose character delicacy certainly was not a prominent ingredient, ascertained that they possessed the power of making noises like knocks, with their knees. Of course, this kind endowment of Dame Nature was regarded by them as a money-making affair, the amassing of dollars being commonly conceded by their kidney to be the final cause of human existence. So they beat about for a route whereon to issue upon their "mission." The spiritual line suggested itself, and they boldly pushed forth after the golden fleece of the sheep that might consent to be herded by them.

Now, in all intellectual villages, and, indeed, in intellectual circles generally, there are always a few enthusiastic investigators who are ever ready to rise above vulgar prejudices, especially that absurd and antiquated one usually entitled common sense. A number of scientific individuals of this class soon formed a clique about the Fish and Fox, and proceeded to enlighten the world upon the philosophy of these new and wonderful phenomena. This was as clear as mud to them, and they managed to make it equally intelligible to the community.

The dire contagion spread. Parsons soon began to dabble in these forbidden mysteries, and with their usual sagacious insight into natural laws, they united magnetism and electricity, of which they knew nothing, with spiritual manifestations, of which they knew less. The newspapers took it up, and sapient editors discoursed upon it with their accustomed ignorant omniscience. Schoolmasters, too, scientific *ex-officio*, as everybody knows, took up the same important question. Even doctors, we blush to write it, doctors, individuals at any rate who wrote M. D. after their names, mumbled strange incomprehensibilities about magnetism and biology. So the people, misled by all their guides, went mad in brigades, and the two female Barnums pocketed numerous dollars.

After a time some *physicians*, who were not merely M. D's, appeared upon the scene. Dr. Austin Flint, one of the most patient, candid and sagacious inquirers belonging to the profession, found the matter assuming an aspect sufficiently grave to demand his intervention. With his usual cautious induction, he accumulated a number of facts to prove that other women could and did produce with their knees the identical noises which so astonished the world when issuing from the neighborhood of these successful imposters. The next step was to place the two women in a position to throw their knees *hors du combat.* This was done, and forthwith the spirits became silent.

The imposture was now fully exposed, and Dr. Flint and his colleagues having satisfied themselves, published to the world the results of their inquiries. Unprejudiced minds were satisfied, but the poor gulls, who enjoyed the palpable hoax played off upon them, were, as might have been expected, indignant at the friends who unmasked the gross impostors. No one should have been surprised at this. People generally have a natural leaning towards the marvelous, and almost always prefer a falsehood to the truth.

"The lover may
Distrust the look that steals his soul away,
But faith, fanatic faith, once wedded fast
To one dear idol, hugs it to the last."

So the honest doctor and his coadjutors were environed by a clamor not less noisy nor more discriminating than that which bellowed in olden days around the fair Latona.

The Fish and Fox, moreover, unwilling to lose even that small section of their followers, who retained sufficient common sense to perceive that black could not be white, resorted to farther experiments, and, after numerous secret rehearsals, succeeded in developing the same class of sounds by snapping their toes. They then had another committee appointed, who placed them in the same position selected by Dr. Flint, which deprived them of the use of their knees only, but not of their toes. Of course, the same sounds were heard, and Dr. Flint, to the great joy of the noodles, was declared to be mistaken. The weak spirits, who had began to open their eyes, closed them again, and resubmitted themselves to the guidance of the fatal sisters.

After a while, the madness which was at first endemic in the centre of the Empire State, soon became epidemic. The mesmerists and biologists had prepared the way for it; they were the shabby John Baptists of this new Barnumistic gospel. The electric telegraph mixed itself up strangely with the spirits, and some dim references to the magnetic theory of the mesmerists were visible in the phenomena of spiritual manifestations.

But everybody who desired to humbug the public had not been so favored by nature as the two successful sisters. It was necessary, therefore, to find some other medium of communication. Then some bright genius discovered that if four people laid their hands long enough upon a light table, that article of furniture would tilt and knock upon the floor. Of course, spirits must have done this. There was, to the minds of these ghostly counsellors, no other way of accounting for the phenomena of table-tipping. It was of no avail to suggest the possibility of some physical cause. They were deaf to reason, impenetrable to common sense. So the table became a sort of snob tripod, and the sharps and flats who sat around it took the place of the ancient priests, as interpreters of these mysterious secrets of cabinet ware.

After a time, as the influence of the humbug became more extensive, new machinery was introduced. Writing and speaking media made their appearance upon the stage. Possessed by these new "*incolœ Pythii,*" their muscles were violently agitated, and they scribbled wild nonsense which their friends regarded as unveilings of the profound mysteries of another world.

In this condition, spiritualism now exists. The old tricks have been generally abandoned on account of the limited number of people who were gifted with the peculiar faculty possessed by its first apostles. The table was always ready, and any one who was disposed to impose on himself or others, could either fancy or pretend to fancy himself a writing, seeing, hearing or speaking medium.

The delusion, too, is spreading. It is one of those remarkable epidemics of insanity which occasionally prostrate whole nations under its baneful influence. It is on account of its hygeinic relations alone that we devote any attention to it. From any other point of view, we could not consent to look at it. We should feel as if we owed an apology to our readers for the space we took up in the consideration of such a barefaced fraud, were it not that recent circumstances had invested it with a gravity of interest, which, alone and unaided, it could never have excited in any sound mind.

The truth is, that hosts of ignorant and unprincipled charlatans are making use of this powerful machinery to enslave many human hearts, and to inveigle many simple spirits. The moral effect has already been frightful. Many a dark deed has been made to look glorious, many a vile scheme has been commended even to pure hearts by the potent influence of sham spirits. Many a villain has succeeded in carrying out his nefarious designs by calling to his aid this monster superstition.

Nor is this all. Even when this machinery is worked, as it sometimes is, by honest well-intentioned self-deceivers, it is productive of scarcely less mischief, though of a different character. People have gone mad over it, and in wild desperation have wrecked their own lives upon its Plutonian shore.

Hence, it demands the attention of every philanthropist, while to the medical philosopher, it presents an object of study of peculiar and absorbing interest, for the reasons which we have already mentioned.

To the medical man, also, there is another consideration of no little consequence. What shall be the therapeutical management of this frightful epidemic? To this a most desponding answer must be given. Those already attacked with it are in a condition well nigh hopeless. We can only have recourse to prophylactic agents. Let us check the spread of the disease, if possible, by fortifying those who are exposed to its influence, but as yet unaffected by it, by a few antidotes derived from common sense. Actuated by these ideas, we propose to devote a few lines to the examination of the pretensions of the self-styled spiritualists, especially to the notions advanced in the singular production, the title of which stands at the head of the present article.

The first trick with which this imposture began, has been sufficiently unveiled. None but the blindest of gulls can be any longer imposed on by this new species of knock-kneed women. The times were, indeed, "sadly *out of joint*," when such things passed for revelations of the profound mysteries of the other world.

But for the table business. "You cannot show," say the spiritualists, "that these phenomena are not dependent upon the influence of spirits. You cannot put your finger upon any well known force of nature which is competent to produce these motions. Baron Reichenbach's od-force has been, by general consent, ruled out as entirely too *odd* to be admitted. So that, as you have not proved, to our satisfaction, that we are wrong, we must of necessity be right." Such is the precious logic which constitutes the substance of spiritualistic arguments, though it is not always put forth in such an honest and intelligible form as we have given it.

The peculiar force of this admirable style of reasoning will be better appreciated by taking another illustration of it. We assume that the moon is made of green cheese. The best authorities, indeed, do not believe it, any more than they believe that spirits move the table. But what do we care for authorities. We believe it, and we have a "mission" to promulgate the doctrine, and to prove it satisfactorily to all unprejudiced minds. As for the Newtons, and Herschels, and Laplaces, and their followers, they are so wedded to their old prejudices, that we give ourselves no concern about them, but address our irresistible arguments to the well known sagacity of the general public, the most enlightened portion of which, of course, consists of our followers. We prove our doctrine by the following incontrovertible train of reasoning. "That the moon is made of green cheese is a most ancient and wholesome doctrine, and *we* endorse it. It is agreeable to our notions of propriety that it should be so. Astronomers, indeed, deny it, but they have not positively proved to the satisfaction of ourselves and our friends of *what* it *is* made. Therefore, it remains perfectly clear and well ascertained that it is made of green cheese." Could anything be more satisfactory ? Is it not the very repetition of the spiritualistic logic ?

The table-tipping attracted much attention, and many were the theories advanced to explain these mysterious movements. Baron Reichenbach's od-force, the non-existence of which had been proved, long before he advanced any hypothesis in reference to it, was brought forward by some. Others while fully admitting all that had been asserted in reference to the action of the tables, attributed their activity to electricity. Of course, no one acquainted with the simplest rudiments of electrical science could assent to this hypothesis. It was the offspring of ignorance alone.

At this time Professor Faraday, beyond all controversy the highest living authority on all matters connected with electricity, appeared upon the stage. He showed the public the absurdity of the various theories which had been advanced to account for these motions, and by a simple but ingenious apparatus, proved that the movements did not originate in the table but in the persons who surrounded it. This result had been confidently expected by those who had any previous acquaintance with recently developed laws in nervous physiology, so well expounded by Dr. Carpenter in the last edition of his famous "Principles." *Expectant attention*, or that condition of the mind in which by earnest looking for a particular motion, the nervous system is so polarized as to act directly upon the muscles and produce the anticipated result, is the true force which lies at the bottom of these phenomena, when they are honestly excited.

So far, then, there is no mystery in these things, none at least which cannot be revealed by the light of physical science. But, say the spiritualists, "how do you account for the intelligent responses given by the furniture when catechized by its attentive pupils?"

We answer first, that we doubt the intelligence of the table. It has amounted to nothing as yet, and is not likely to amount to any thing. It has not added the smallest fraction of a fraction to the sum of our knowledge. It has not revealed the thinnest edge of a new fact. It has been the baldest, flattest, stalest, most jejune repetition of the tritest moral saws. Its revelations have never risen above the level of those who sat around it, nor indeed could it be expected that the block under the hand should be more intelligent than the blocks upon the shoulders.

In the second place, we would say, that in every well authenticated statement of investigations into this business, it has been invariably declared that, whenever ordinary prudence had been observed in the concealment of facts which were known only to the questioner, the table made the most absurd mistakes, showing of course that the manipulators could gain nothing from the furniture which they did not possess themselves.

As for the medium business, swindling is so very easy in that line, that it requires miraculous stupidity to consent to investigate it. The dreary trash which they dribble out—the pompous weary drivel of their revelations is too somniferous for us. Opium, chloroform and all the sleepy drugs of the pharmacopœia could not more effectually benumb our mind. We therefore let that pass.

But really, we are ashamed of ourselves, that we have consented to treat so preposterous a subject with gravity. The revelations themselves are too ridiculous for any thing but Homeric laughter, if it were not for their results. But when people dash their brains out, and go mad about them, it is time to look into the matter.

It was but a short time since that a lady of our acquaintance assured us with the utmost gravity that at the meeting of a spiritual circle, she had summoned the shade of Napoleon into a band box, with which she manipulated, and made him dance the Marseillaise while she sang it. A worthy object, truly, for which to disturb the rest of that mighty spirit.

But many others greater, better than Napoleon are at the mercy of these daring experimenters. They can be summoned from the majestic regions of the dead where they slumber with princes and counsellors, for the most frivolous purposes. Any set of investigating noodles, or garrulous old women who choose to put their hands upon a block as senseless as themselves, can desecrate the sacred solemn repose of the mighty dead. That glorious "rest" which scripture says "*remaineth*," can be broken up at any time by dull heads nodding around as dull a table.

The old superstitions had at least the redeeming merit of poetry and a sense of fitness, of which this is wholly devoid. A spirit was in ancient days, only called from its repose by some powerful motive or some irresistible incantation. The magician must first draw his circle with consummate art, and collect all the materials of his ceremony with the utmost care. Then every thing having been done with the solemnity and gravity befitting so momentous an occasion, the invocation is said, and nothing less than the terrible and sacred name of the Creator which coerces all nature, brings the reluctant spirit from the Silent Land.

And when the modern spirits do leave their spheres, to what purpose do they come? What intelligence do they bring? What do they tell us? Why, that old Mrs. Smith's "rheumatiz" is as bad to-day as it was yesterday, and that it probably won't be any better to-morrow; that Mr. Jones is 77 years of age and had better prepare for another world; that there are several

*spheres* of spirit life, and that all are not the same; that two is not four, and that black can scarcely by an unprejudiced mind be mistaken for white. Then they throw matches about, and knock over furniture and slam doors, and tie pocket handkerchiefs in knots. We are gravely informed that Daniel Webster knows how to count, and that Henry Clay has not forgotten his multiplication table. To get off this and similar twaddle, these great spirits can consent to hammer out their dull revelations with a table leg. Who could imagine a slower or more stupid way for the quick intelligence of disembodied spirits to communicate with those they have left behind them. Those vivid intellects, that dart through time and space with such incredible speed, must stop to spell out in slow raps their messages. It is too absurd for any one out of the idiotic wards of an insane asylum to believe.

But it is time to come to the delectable composition which has excited this somewhat discursive commentary. As the title shows, it is the joint composition of a judge and a doctor, assisted, as they say, by the spirits of the departed, chiefly of Swedenborg and Lord Bacon. What the doctor's antecedents in this spiritual matter may have been, we cannot tell, but we gather from the book itself that the judge's mind was laboring under a severe affliction, which seems to have laid him open to hallucinations, and predisposed him to the influence of these singular errors. The evidence upon which his belief is founded can scarcely require any attention here. It is a mere repetition of the raps, writings, and revelations which have become such trite and common things.

Of Dr. Dexter's antecedents, as we have said, we know nothing. We never heard of him before, until his name was mentioned in connection with these assumed revelations. His *rôle* is that of a sceptic gradually converted to the truths of spiritualism, and the *evidences* are of the same nature of those which wrought conviction on the spirit of the judge. We pass these things by, and shall endeavor to ascertain what ideas of spirits and spiritual things this book contains.

In the first place, the absurd electrical theory is glanced at rather than distinctly proposed. A paragraph or two, copied at random from the spiritual statements, will show the ideas entertained by the media.

"You sit in a circle. Now, the material constituents of which the body is composed, are alike in the bodies of men. And when you *sit in a circle, an equilibrium of the magnetic forces is established,* for electricity or magnetism exists in everything on earth, either in one condition or another. When by sitting, the equilibrium is established, then some one is selected whose *nervous system* is most easily controlled by the exercise of one will. I stand near him. * * * I establish a *concurrent simulation* with his nervous system, and thus have the control."

"When there is an accordance between two minds on earth, it increases the electric affinities, and makes easier the power to communicate."

"When there is an interruption to the full flow of the electrical current, and an *entire absence of passiveness* in the mind of the medium, it prevents communications, and at the same time *develops another principle* that acts antagonistically to the spirit influence. It becomes very important, too, that the minds of the circle should be directed to the subject discussed by the spirits, so that the nervous properties may be readily seized."

Now, the absurdity of these notions, and the remarkable ignorance of electrical laws and physiological science evinced by them, is too palpable to escape the notice of the merest tyro in these studies. The silly fancies of travelling mesmerists, and the exploded ideas of the electrical character of nervous influence constitute the staple of their theory.

If to these electrical notions be added the extraordinary one, to be found on page 169, the wonder of the reader will be excited to know what sort of ideas of physical science that man must have who could believe these two statements. We are there told that the evil spirits, after having passed into new bodies, are so heavy, "so much more dense than the other spirits, that they cannot maintain themselves even near the earth, but *sink far below it,* and are really of so dark a hue, that they are almost black." These ideas of gravitation belong to an age far more remote than that of Swedenborg, who is said to utter them.

The spirits who communicate with the two new prophets are Swedenborg and Lord Bacon. The latter assures them that his "nature has somewhat *progressed* since he left the earth," that he is not "that dull, matter-of-fact spirit *as* he was when a man on earth, &c." The Baconian language has certainly changed. That pure, lofty, Elizabethan English of the living Chancellor has sadly suffered. Not only does he indulge in such vile *patois* as that which we have just quoted, but gives utterance to the most infamous English. Thus he covers such adjectives as "*fartherest*," and is guilty of the most atrocious massacre, not only of the Queen's English, but even of the President's American.

One of the most amusing instances of a spiritual dodge occurs on page 113. They were conversing on the truth of Bacon's statements especially in reference to his identity, when the judge remarked that if he was *truthful* in other respects he would regard him as such in reference to his identity, and that he had in his mind a law maxim which he would like Bacon to write. The spirit answers that he cannot exactly say, but thinks it to be "that you are bound to believe every thing to be true until proved false." The judge replies "no, it is the very reverse." (*Falsus in uno, falsus in omnibus.*) The spirit then answers, "do you not know that when you try this test, you set on the doctor's mind itself to solve the question?" and adds, that he has not thought of earth's law for past a century.

The total failure of the judge to detect or even to suspect this manifest prevarication renders it impossible to place any reliance upon his powers of sifting and examining evidence of a physical character. Dr. Dexter's guess was a very natural one under the circumstances, but he failed to make a successful retreat and so retrieve the honor of his protege.

The religious sentiments of the book do not properly fall under our notice. It is sufficient for us to say that there is nothing at all new in them. They are only the old deistical and pantheistical notions, not even remodelled. The Mosaic account of the fall of man and the Evangelists' history of the incarnation are both denied upon the old grounds, and the opinions advanced on these subjects would not require any examination from any quarter.

At the end of the book are fac-similes of the signatures and manuscripts of the various spirits who used Dr. Dexter's hand to write their thoughts. Prefixed to this is one of Dr. Dexter's notes to the judge. Now, though the general appearance of these different specimens of chirography seems at first glance to be very diverse, a careful examination will show a very close resemblance among them. A comparison of individual letters will satisfy the unprejudiced inquirer that they are all the same hand, very imperfectly disguised.

The general impression derived from the perusal of the book is, therefore, the conviction that it is a tasteless *rechauffée* of fragments of old and dead philosophies, and that it contains nothing of the slightest value or interest to any person of sound mind.

As to its style, it is very bad. It is at once jejune and stilted. An extreme paucity of ideas with an uncommon quantity of words is always disagreeable, but when that is added to a diction at once pompous and vulgar, which ignores all the laws of grammar and rhetoric, it becomes something more than merely tiresome. It was extremely injudicious in the doctor to select Bacon as one of his interlocutors, because the contrast between the stately, thoughtful, but simple style of the real Chancellor, and the pompous inanities of his counterfeit, is too palpable, and too easily made.

